# Supplementary material for: Cell-of-origin and genetic drivers define advanced bladder cancer subtypes and potential therapeutic response in mouse models
Source: J Exp Clin Cancer Res. 2026 Feb 23;45:79. doi: 10.1186/s13046-026-03677-8 (PMC13032214; doi:10.1186/s13046-026-03677-8)
Supplement: Supplementary file 2 — Supplementary Material 2. [file 13046_2026_3677_MOESM2_ESM.pdf]

"Cell-of-origin and genetic drivers define advanced bladder cancer subtypes and potential therapeutic response in mouse models"

Authors: Ester Munera-Maravilla[1,2,3], Mercedes Pérez-Escavy[1,2,3], Carolina Rubio[1,2,3], Cristina Segovia[1,2,3], Iris Lodewijk[1,2,3], Sandra P. Nunes[1,2,4], Álvaro Martín de Bernardo[1,2], Ignacio A. Reina[1,2,3], Esther Montesinos[1,2], Lucía Morales[1,2,3], Víctor G. Martínez[1,2,3], Ainara Álvarez-Prada[1,2], Mónica Martínez-Fernández[1,2,3], Marta Dueñas[1,2,3], Jesús M. Paramio[1,2,3], Cristian Suárez-Cabrera[1,2,3]

1. Cellular and Molecular Oncology and Genitourinary Tumor Group. Institute of Biomedical Research, Hospital Universitario 12 de Octubre, Madrid, Spain.
2. Molecular and Translational Oncology Division. Centro de Investigaciones Energéticas, Medioambientales y Tecnológicas (CIEMAT), Madrid, Spain.
3. Centro de Investigación Biomédica en Red de Cáncer (CIBERONC), Madrid, Spain.
4. Cancer Biology and Epigenetics Group, Research Center of IPO Porto (CI-IPOP)/CI-IPOP@RISE (Health Research Network), Portuguese Oncology Institute of Porto (IPO-Porto)/Porto Comprehensive Cancer Center Raquel Seruca (Porto.CCC), Porto, Portugal.

Authorship note: EMM and MPE contributed equally to this work (See author contributions). JMP and CSC are co–corresponding authors.

SUPPLEMENTAL FIGURES

This file contains:

Supplemental Figures 1-16

Supplemental Figure 1

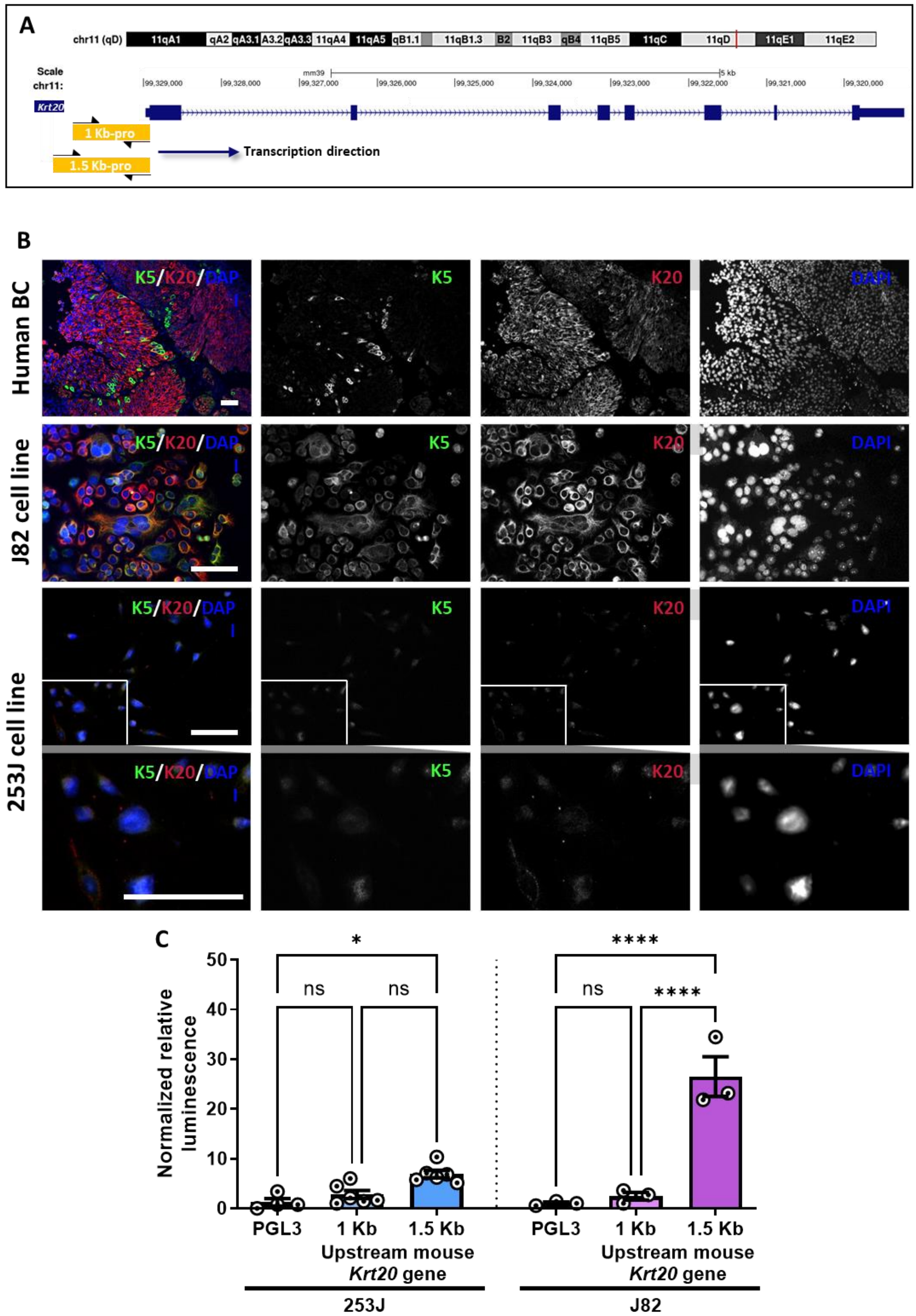

Supplementary Figure S1. Cloning and *in vitro* characterization of murine *Krt20* promoter. A. Selected sequences of 1 Kb and 1.5 Kb (shown as yellow rectangles) upstream of the start codon of the murine *Krt20* gene (located in Ch11qD; marked with a red line). The scheme of the mouse *Krt20* gene is shown; the thin parts of the element represent untranslated regions (UTRs), whereas the thick parts represent exons. The scale is in kilobase pairs (Kb). **B.** Immunofluorescence assays to evaluate the expression of keratins K5 (green) and K20 (red) in a human BC tissue and in human BC cell lines. Nuclei are stained with DAPI (blue). Scale bars = 200 µm. In the last row, a high magnification of 253J cells is shown. **C.** Luciferase assay results to evaluate the promoter functionality of the cloned sequences (represented in figure A) in the PGL3 plasmid in both 253J and J82 cell lines. Mean ± SEM and individual values are shown. ns = not significant; *p-value< 0.05; ****p-value< 0.0001.

Supplemental Figure 2

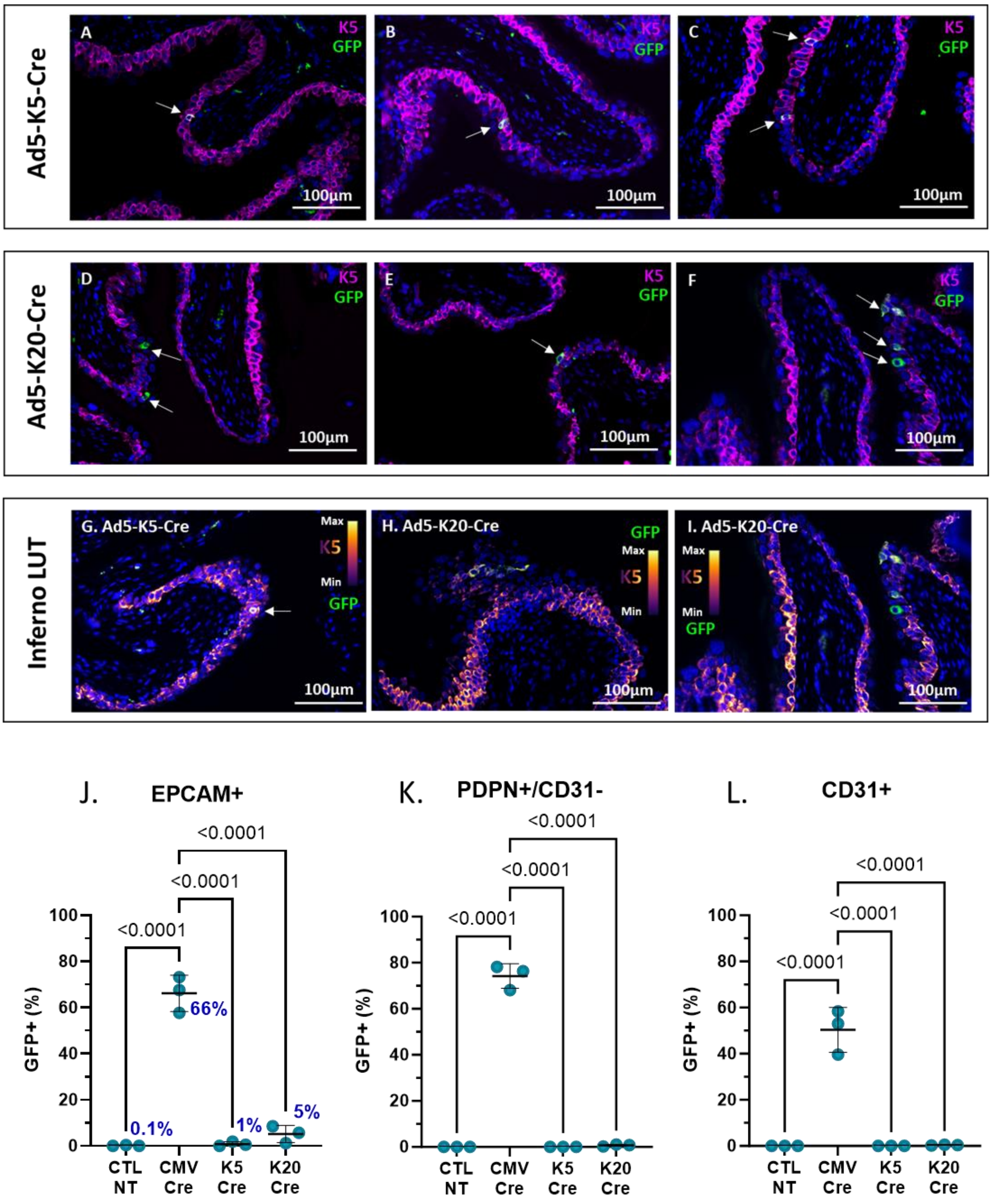

Supplementary Figure S2. *In vivo* and *ex vivo* analysis of adenoviral promoter specificity and cellular targeting. **A-F.** Representative images of keratin 5 (pink) and GFP (green) co-staining in the bladder urothelium of *mT/mG* reporter mice, showing GFP⁺ cells in the basal layer following Ad5-Krt5-Cre intravesical injection (A-C; upper panels) and in the luminal layers after Ad5-Krt20-Cre administration (D-F; lower panels). **G-I.** Keratin 5 expression is displayed using the Inferno lookup table (LUT), with intensity values ranging from high (yellow) to low (dark purple). GFP⁺ cells are shown in green; co-expression appears in white. Arrows indicate GFP⁺ cells. Scale bar = 100 µm. Note: Green signal detected outside the epithelial compartment corresponds to erythrocyte autofluorescence. **J-L.** *Ex vivo* analysis of adenoviral targeting. Bladders from *mT/mG* mice were dissociated, cultured, and transduced with adenoviruses (Ad5-CMV-Cre, Ad5-Krt5-Cre, and Ad5-Krt20-Cre). Flow cytometry plots show the percentage of GFP⁺ adherent cells within epithelial (EpCAM⁺; J), mesenchymal fibroblast-like (PDPN⁺/CD31⁻; K), and endothelial (CD31⁺; L) populations. Data are presented as mean ± SD from three independent bladders, with each bladder shown as an individual data point. For epithelial cells, mean values are highlighted in dark blue. NT = non-transduced. ****p < 0.0001.

Supplemental Figure 3

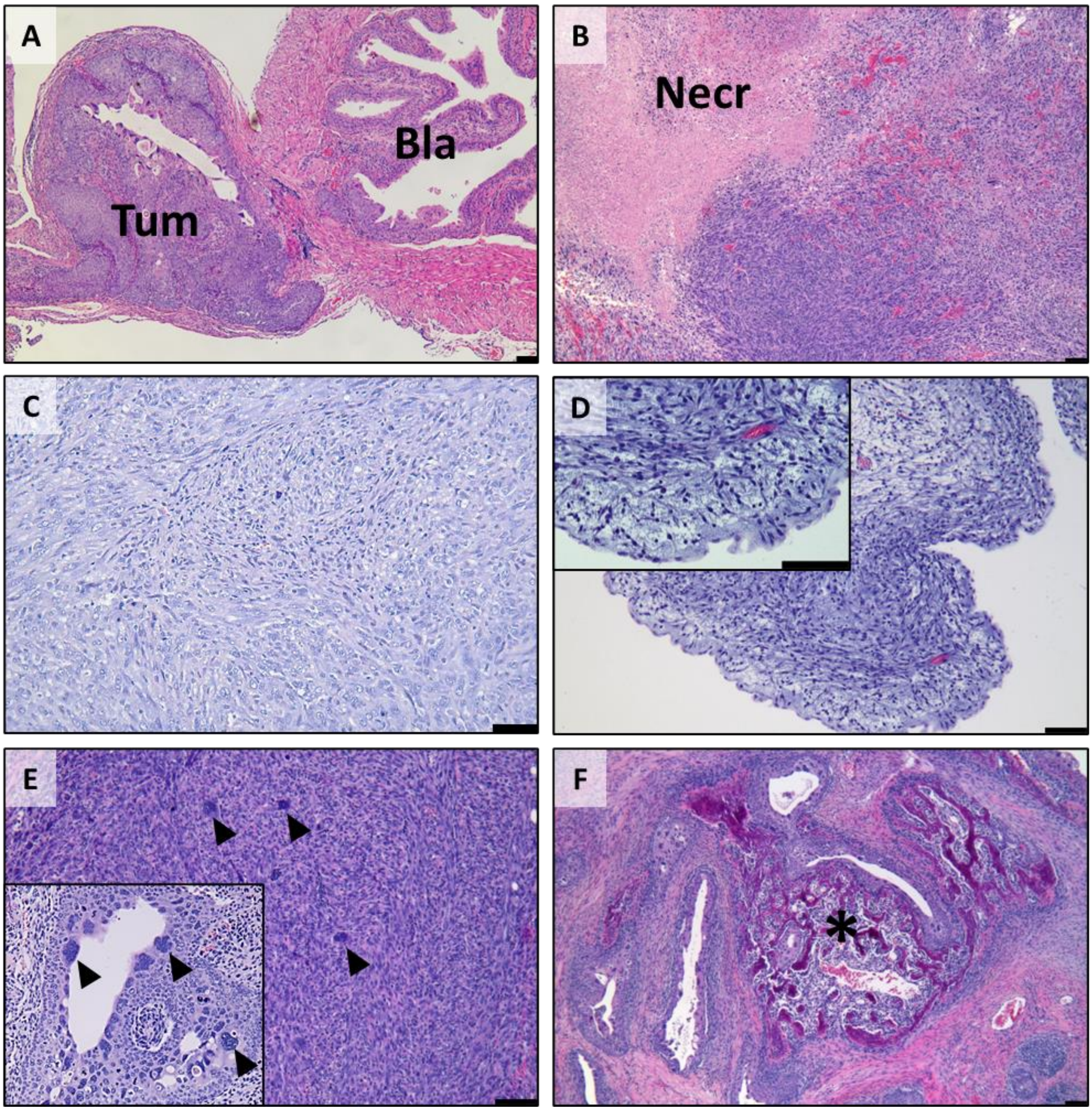

Supplementary Figure S3. Histological characteristics of tumors from the different BC mouse models. A. Invasive tumor arising from the bladder in a QKO mouse. **B-F.** Representative H&E staining showing the most common histological features of tumors in the various models, including necrosis (B), immune cell infiltration (C), myxoid stroma (D), giant pleomorphic cells (E) and bone metaplasia with calcification (F). In D, a high magnification is shown. In E, the insert shows giant pleomorphic cells also in a differentiated tumor. Scale bars = 200 µm. N = necrosis; head arrows = giant pleomorphic cells; * = bone metaplasia; T = tumor; B = bladder.

Supplemental Figure 4

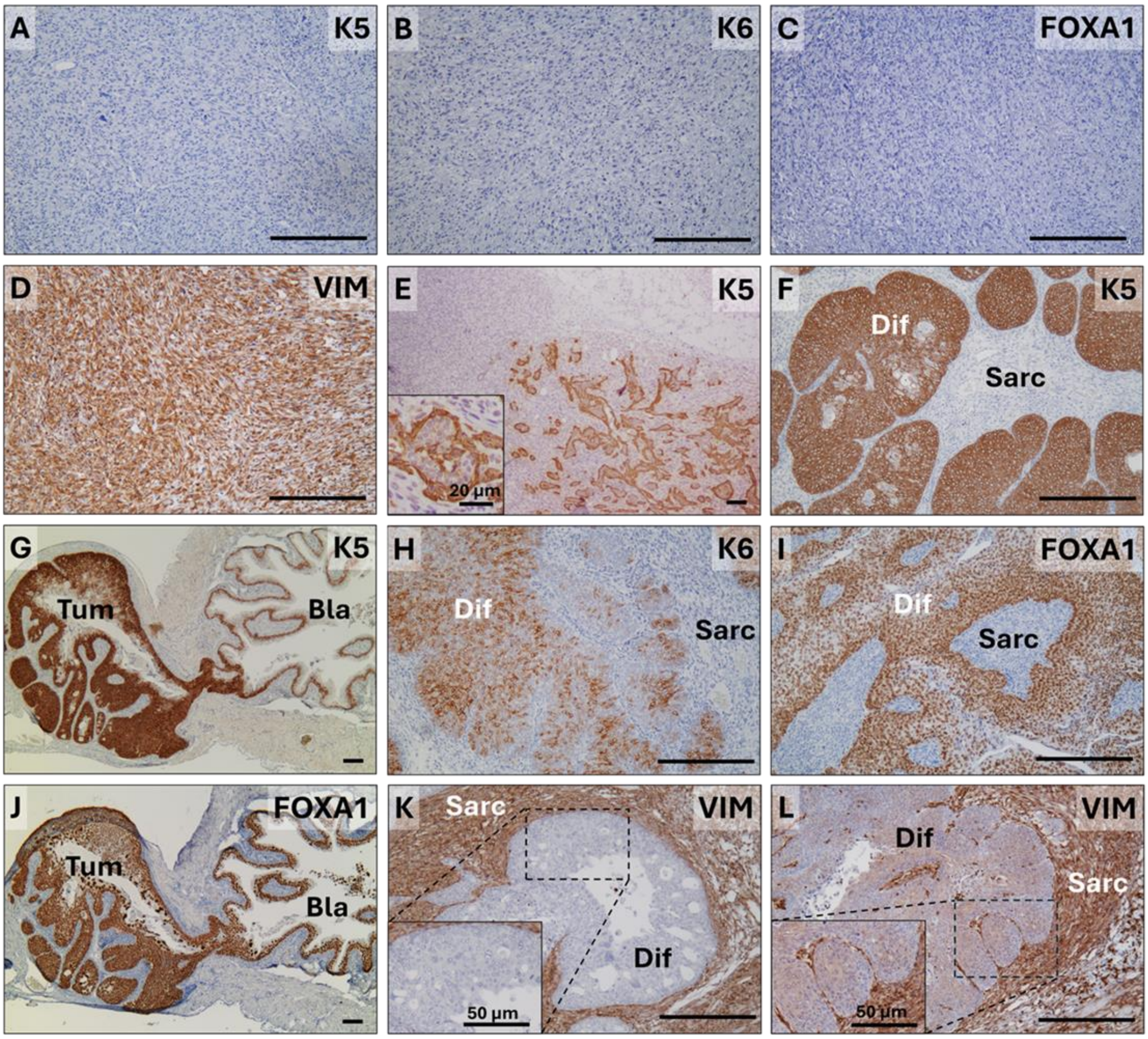

Supplementary Figure S4. Immunohistological characterization of tumors from different BC mouse models. A-D. Representative immunohistochemical images of dedifferentiated tumors showing staining for keratin 5 (K5; A), keratin 6 (K6; B), FOXA1 (C), and vimentin (VIM; D). **E.** Keratin 5 expression in distinct regions of a dedifferentiated tumor arising in DKO-K5 mice. **F-K.** Representative immunohistochemical images of differentiated tumors showing staining for keratin 5 (K5; F and G), keratin 6 (K6; H), FOXA1 (I and J), and vimentin (K and L). High-magnification images are shown in panels E, K, and L. Scale bars = 100 µm, unless otherwise indicated. Sarc = sarcomatoid pattern; Dif = differentiated pattern; Tum = tumor; Bla = bladder.

Supplemental Figure 5

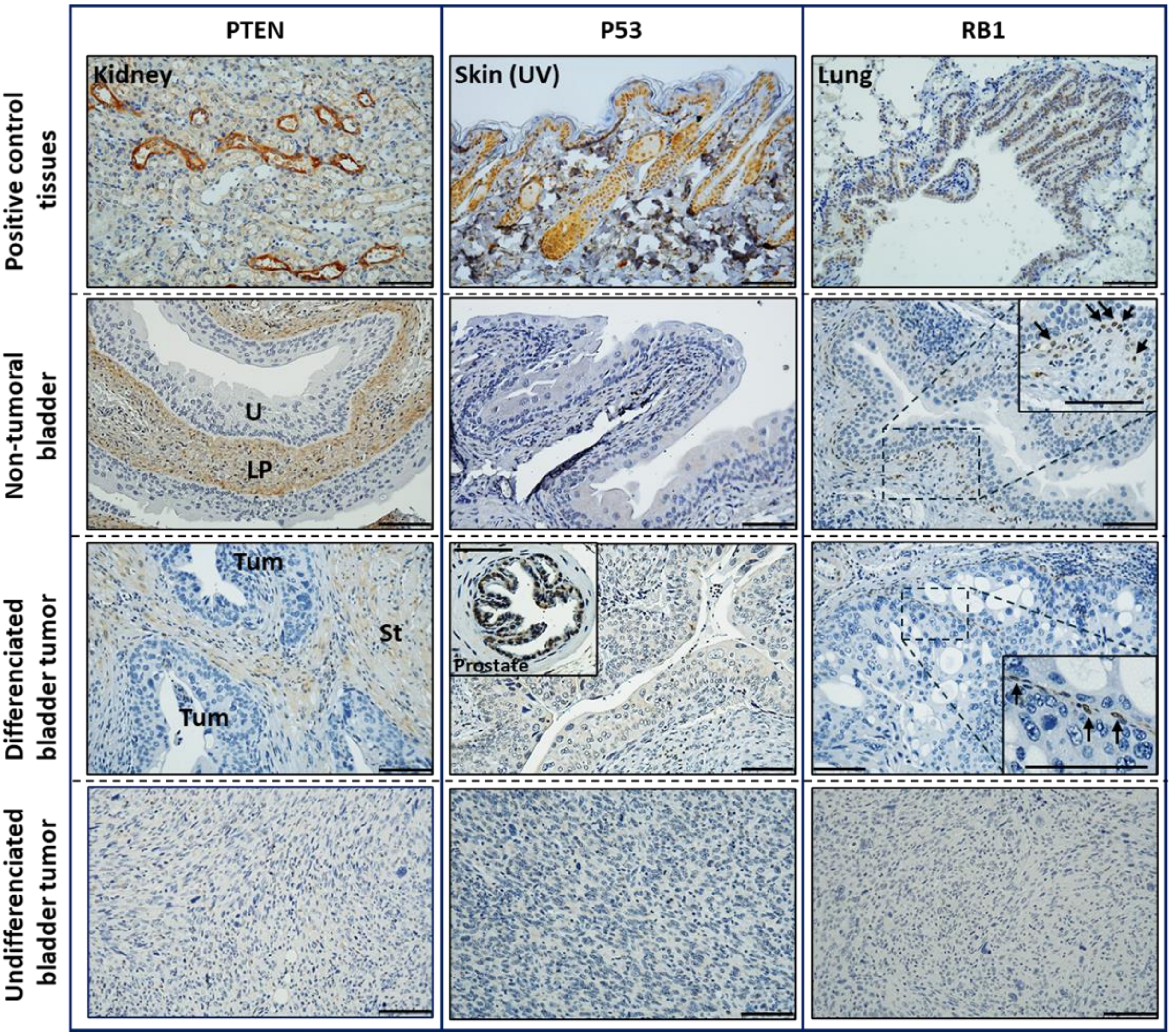

Supplementary Figure S5. Validation of protein loss or deletion in tumors generated by the models. Immunohistochemical staining for PTEN (left panels), p53 (central panels), and RB1 (right panels) showing expression in positive control tissues (kidney for PTEN, UV-irradiated skin for p53, and bronchus for RB1), as well as in normal bladder and in differentiated and dedifferentiated tumors. Arrows indicate RB1-positive nuclei in stromal cells in selected high-magnification images. In the p53 staining of differentiated tumors, a prostate gland present in the same histological section is shown as an internal positive control. U = urothelium; LP = lamina propria; Tum = tumor; St = stroma. Scale bar = 100 µm.

Supplemental Figure 6

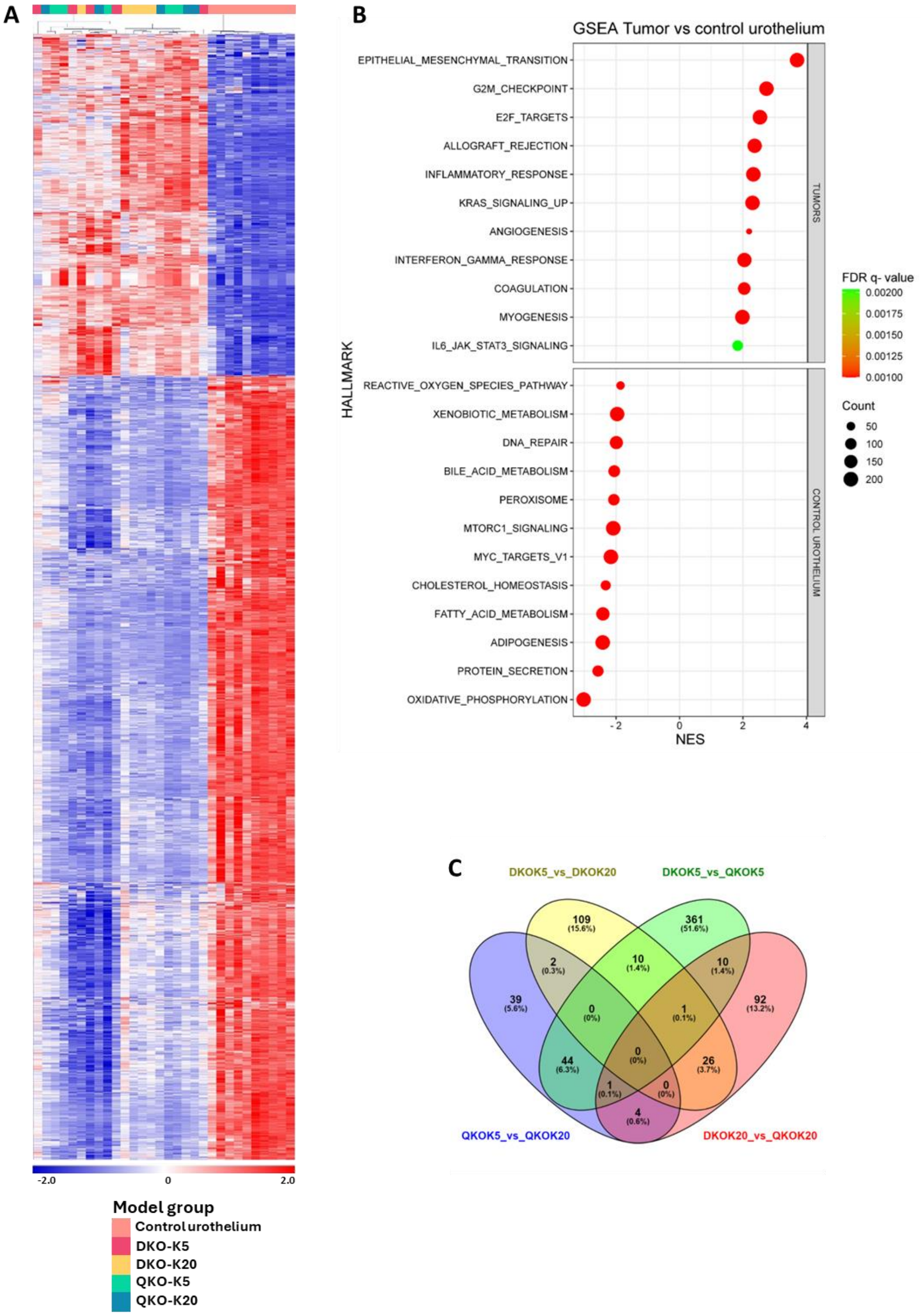

Supplementary Figure S6. Transcriptomic analysis comparing tumors from the different mouse models with normal urothelium. A. Heatmap illustrating the clustering of differentially expressed (DE) genes between control tissue and tumors (fold change ±3 and FDR p-value < 0.00005). **B.** Gene Set Enrichment Analysis (GSEA) identifying the most significantly enriched gene sets from the cancer hallmark molecular signature when comparing tumors (top panels) and control urothelium (bottom panels). Normalized enrichment score (NES), FDR q-value, and gene set size (count) are provided. **C.** Venn diagram showing the overlap of DE genes across the different BC mouse models comparisons (fold change ±2 and p-value < 0.05).

Supplemental Figure 7

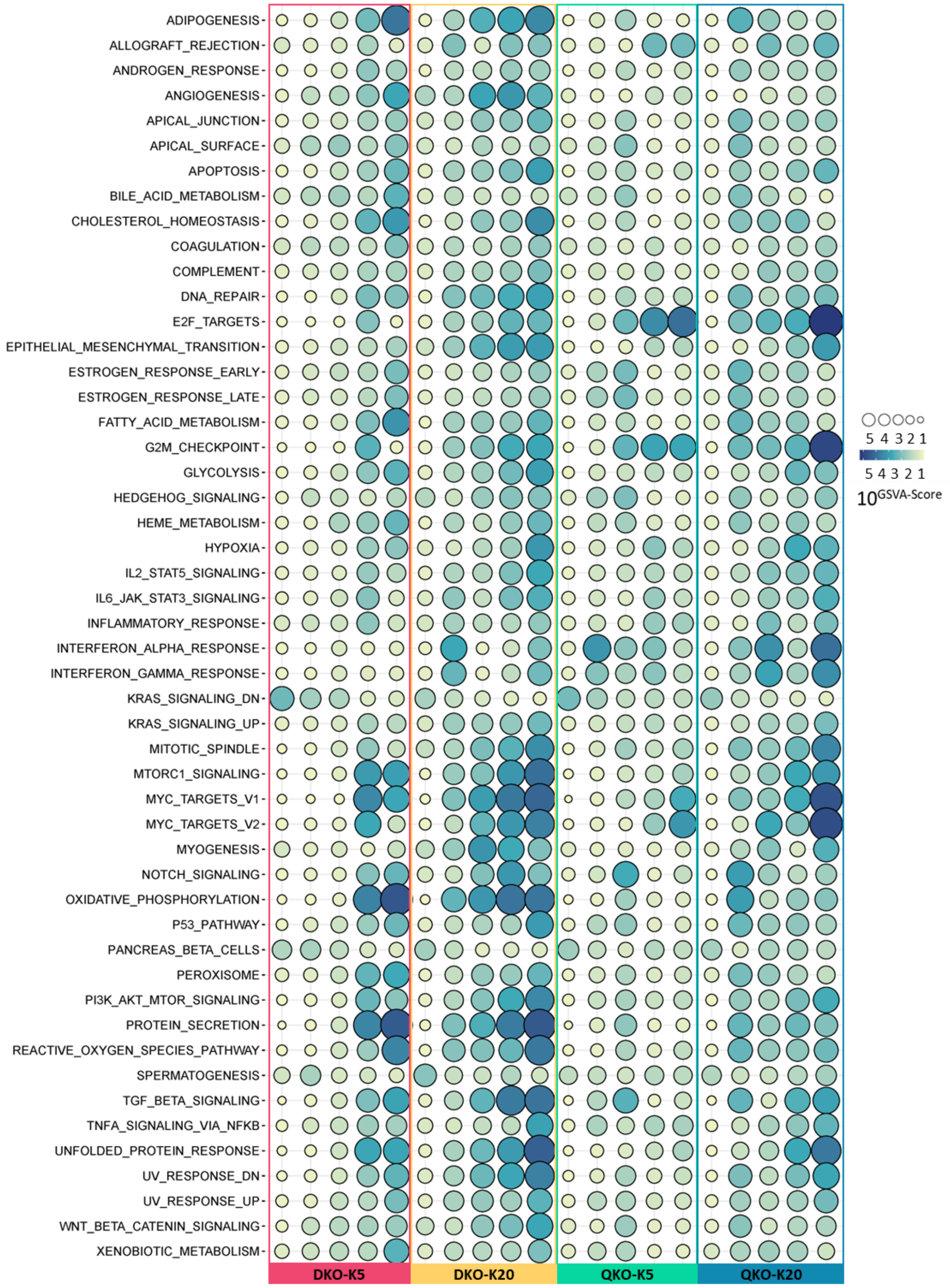

Supplementary Figure S7. Gene Set Variation Analysis (GSVA) scores for tumors across models illustrating all gene sets from the cancer hallmark molecular signature. Scores are presented as $10^{\text{GSVA-score}}$.

Supplemental Figure 8

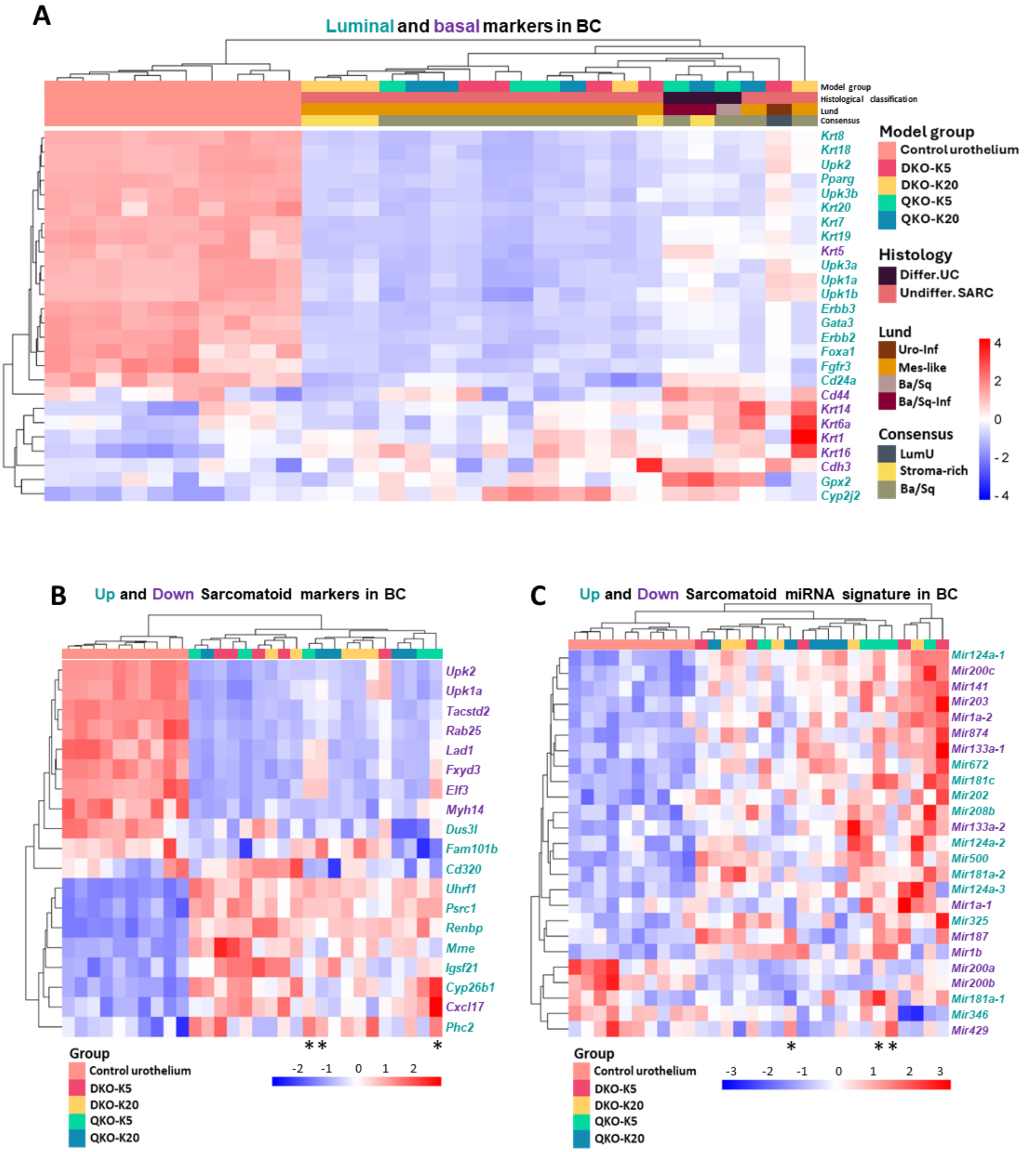

Supplementary Figure S8. Clustering of tumors from the different mouse models according to BC gene signatures. A-C. Heatmaps displaying hierarchical clustering of tumors across mouse models based on: (A) luminal and basal markers of BC, (B) mRNA and (C) miRNA sarcomatoid signature in BC. In A, tumor morphology and molecular classification associations are indicated. Luminal markers are highlighted in blue, while basal markers are shown in purple. In B and C, asterisks denote differentiated urothelial carcinoma; upregulated genes are highlighted in blue, while downregulated genes are shown in purple.

Supplemental Figure 9

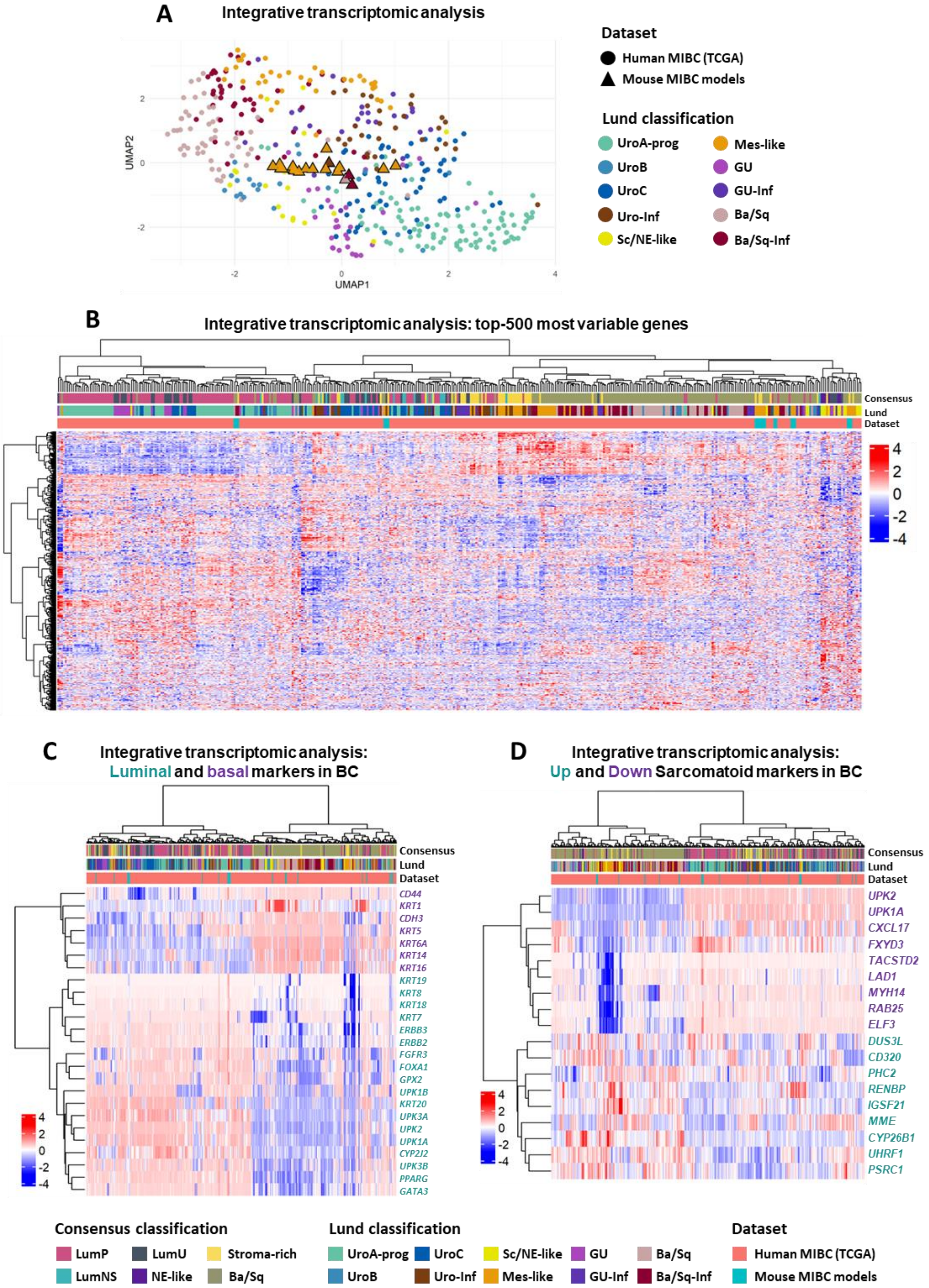

Supplementary Figure S9. Integrative molecular clustering of mouse BC models and TCGA MIBC samples. A. UMAP plot illustrating integrative transcriptomic analysis of tumors from BC mouse models together with MIBC samples from TCGA database, with tumor distribution colored according to the Lund molecular classification. **B-D.** Heatmaps displaying hierarchical clustering of mouse and human tumors based on: (B) the 500 most variable genes, (C) luminal and basal BC signature markers, and (D) a sarcomatoid gene expression signature. Consensus and Lund subtype annotations are included. In C, luminal markers are highlighted in blue, while basal markers are shown in purple. In D, upregulated genes are highlighted in blue, while downregulated genes are shown in purple.

Supplemental Figure 10

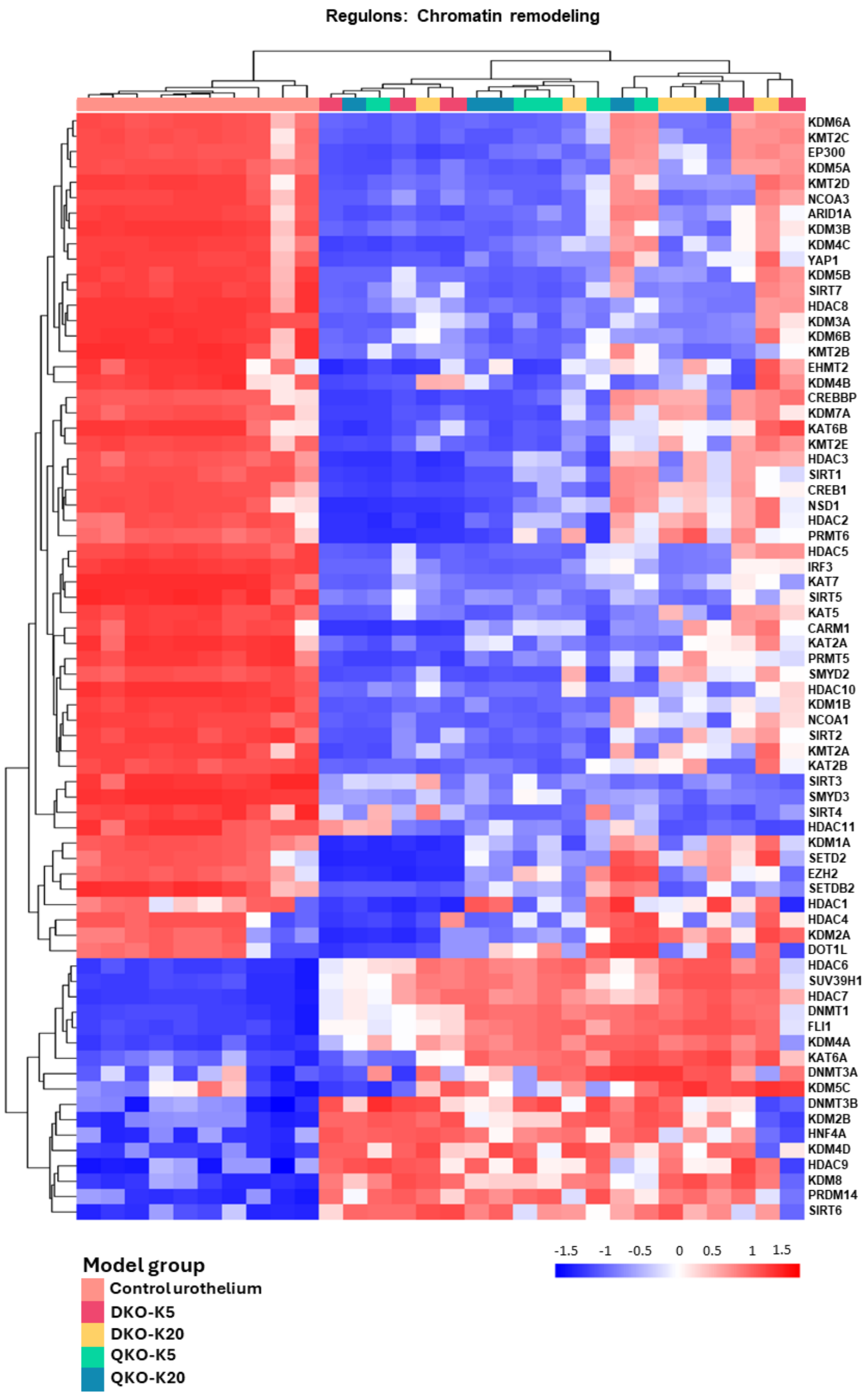

Supplementary Figure S10. Analysis of chromatin remodeling regulons. Heatmaps showing hierarchical clustering of tumors across mouse models based on regulon activity of chromatin remodeling associated with BC.

Supplemental Figure 11

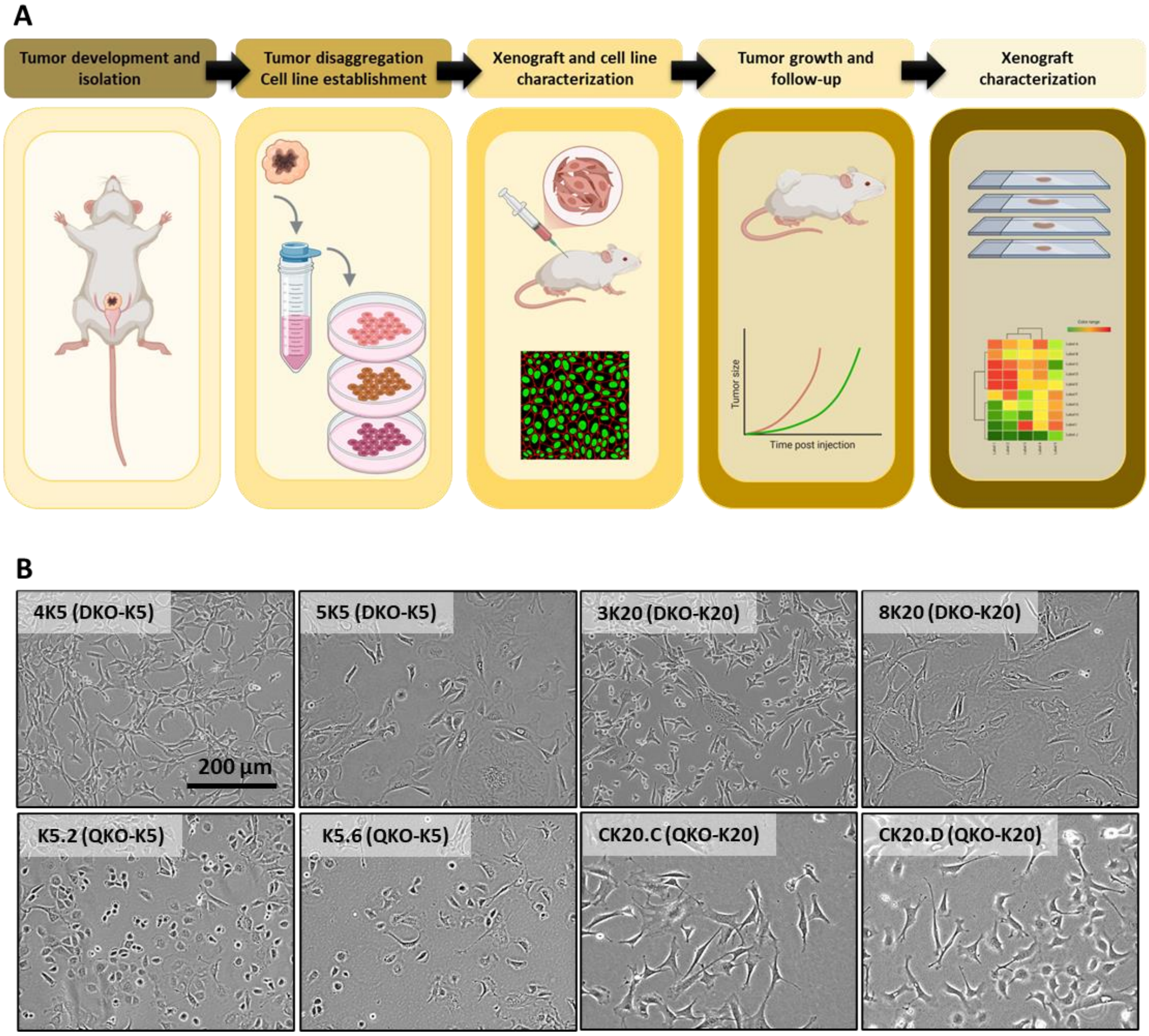

Supplementary Figure S11. Establishment of tumor cell lines from different models. A. Schematic representation of tumor isolation and disaggregation to derive tumor cells lines, followed by their characterization and evaluation of growth capacity in immunocompetent models. **B.** Representative images of some established cell lines showing their morphology.

Supplemental Figure 12

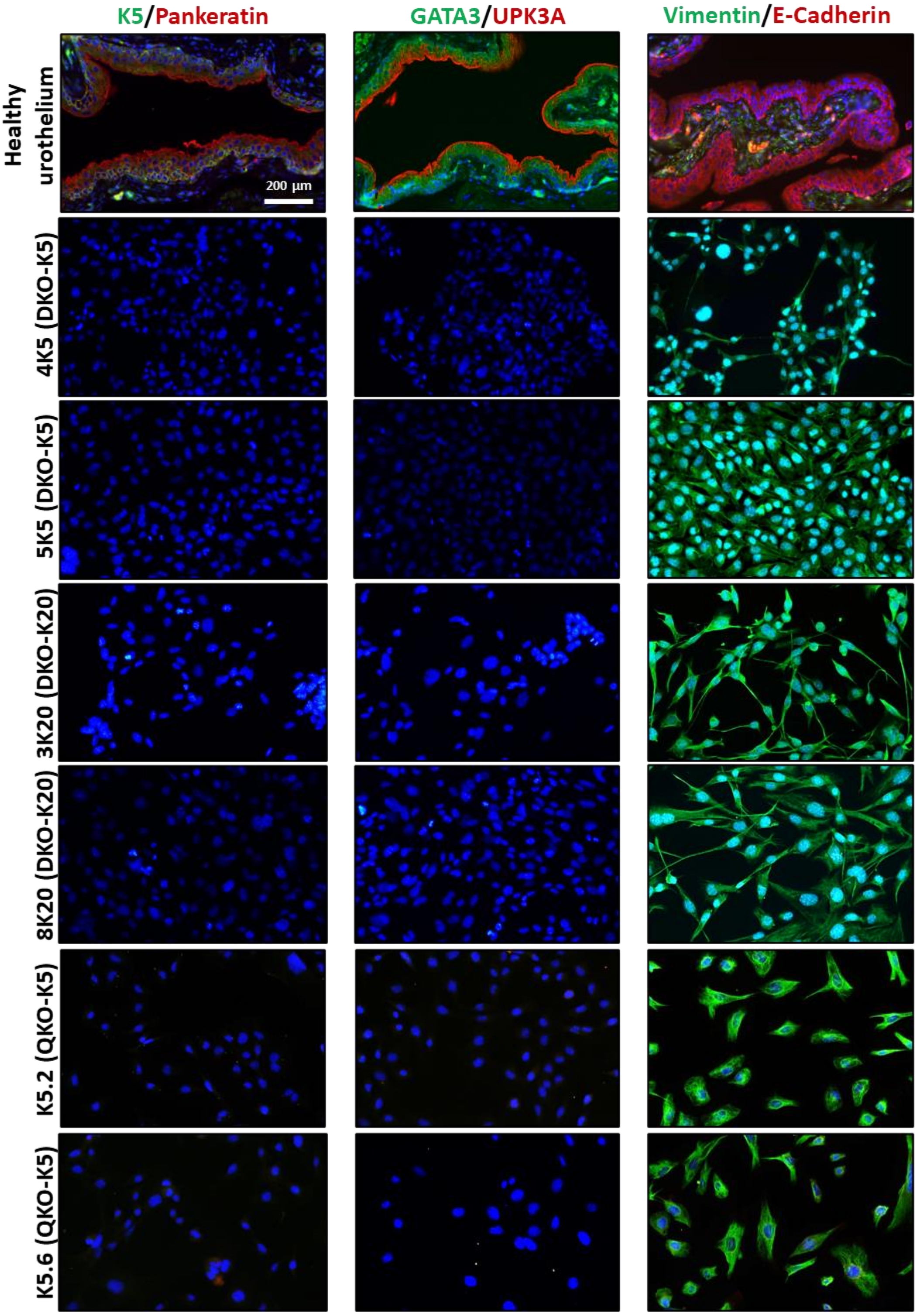

Supplementary Figure S12. Immunofluorescence analysis of epithelial and mesenchymal biomarkers in various established cell lines. Healthy urothelium was included as a positive control. Scale bar = 200 µm. All images were acquired using the same magnification and scale settings.

Supplemental Figure 13

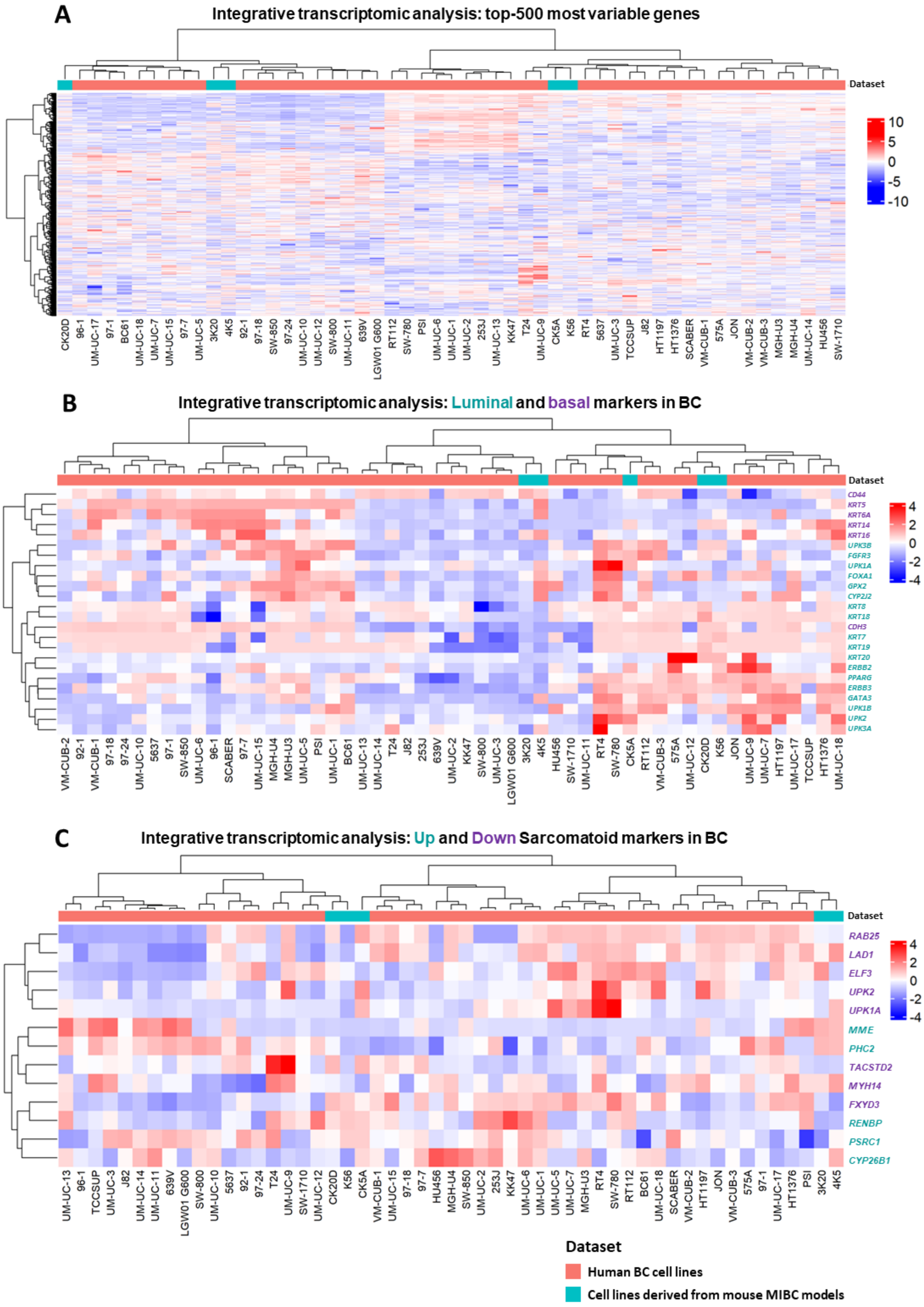

Supplementary Figure S13. Integrative molecular clustering of mouse BC cell lines and human BC cell lines. A-C. Heatmaps displaying hierarchical clustering of mouse and human BC cell lines based on: (A) the 500 most variable genes, (B) luminal and basal BC signature markers, and (C) a sarcomatoid gene expression signature. In B, luminal markers are highlighted in blue, while basal markers are shown in purple. In C, upregulated genes are highlighted in blue, while downregulated genes are shown in purple.

Supplemental Figure 14

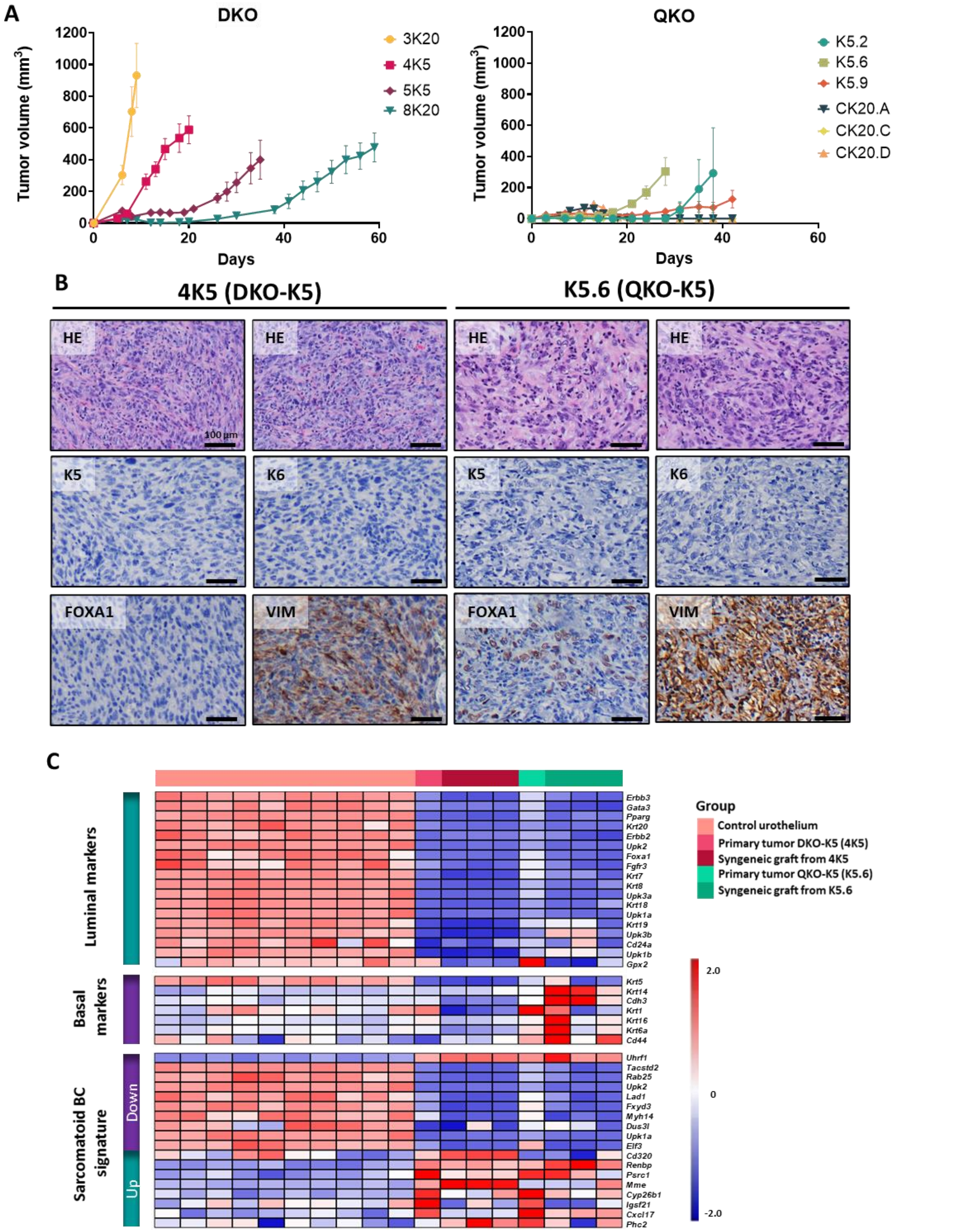

Supplementary Figure S14. Characterization of immunocompetent syngeneic graft BC mouse models. A. Tumor growth following heterotopic injection of established cell lines (DKO and QKO) into immunocompetent mice. Data are presented as mean ± SEM. **B**. Representative images of tumors formed after injection of 4K5 (DKO-K5) and K5.6 (QKO-K5), showing tumor morphology (H&E staining) and the expression of luminal (FOXA1), basal (K5/K6), and mesenchymal (vimentin) markers analyzed by immunohistochemistry. **C.** Transcriptomic analysis of bladder tumors derived from immunocompetent syngeneic grafts, compared to their primary tumor of origin, using luminal, basal, and sarcomatoid gene signatures from human BC.

Supplemental Figure 15

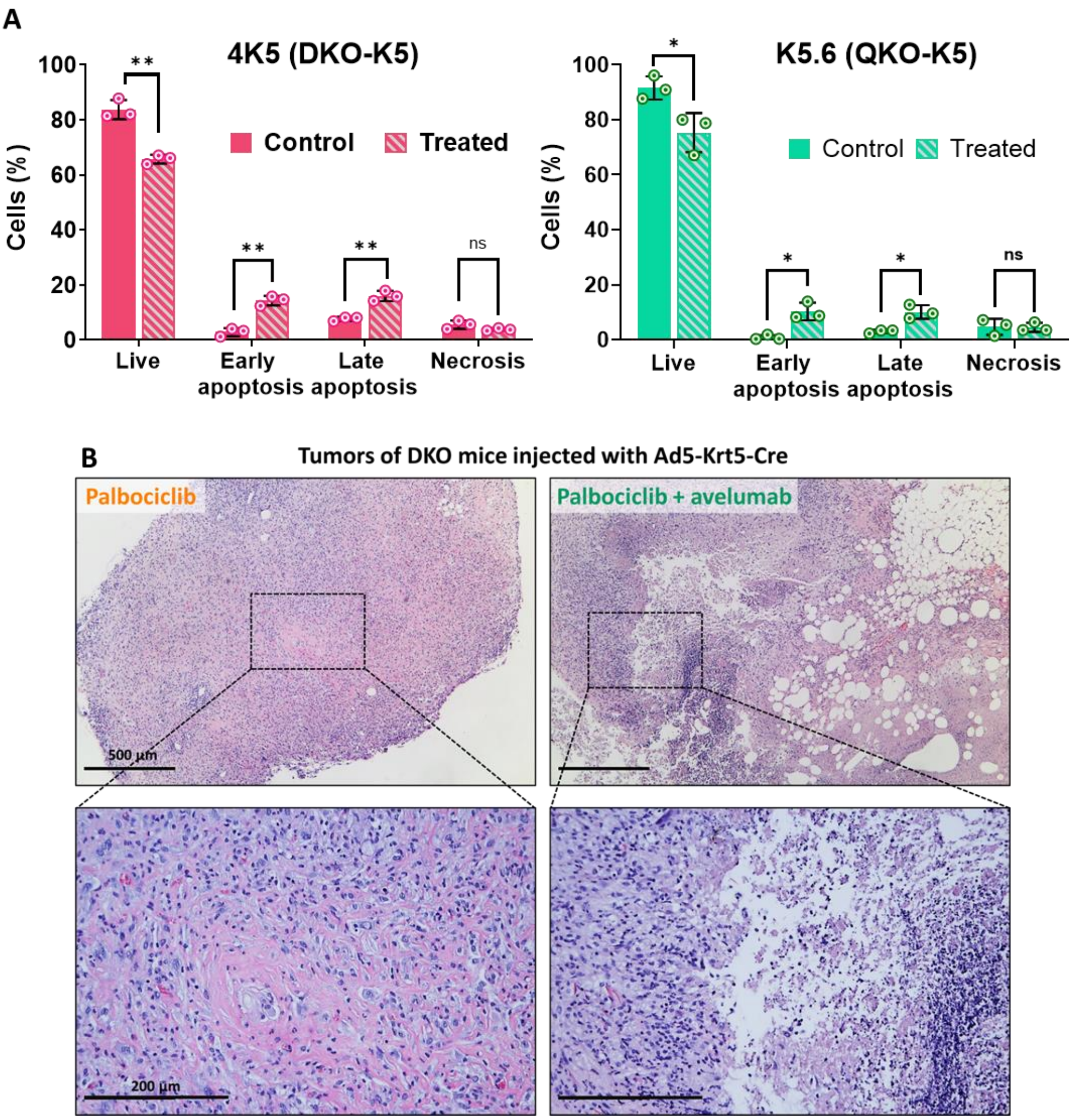

Supplementary Figure S15. *In vitro* and *in vivo* analysis of tumor cell response to treatment. A. *In vitro* analysis of apoptosis and cell death in mouse BC cell lines derived from K5-positive cells isolated from DKO (4K5; left) and QKO (K5.6; right) tumors following 48-hour treatment with palbociclib at the $IC_{50,}$ analyzed by flow cytometry. Data are presented as mean ± SD from three independent experiments, with the average value of each experiment shown as a single data point. **B.** Proof-of-concept experiment with palbociclib alone (n = 5) or combined with avelumab (n = 5) in DKO mice injected with Ad-K5-Cre into the bladder lumen. A control group of 5 mice was used. Representative H&E-stained tumor sections are shown post-treatment. ns = not significant; *p-value< 0.05; **p-value< 0.01.

Supplemental Figure 16

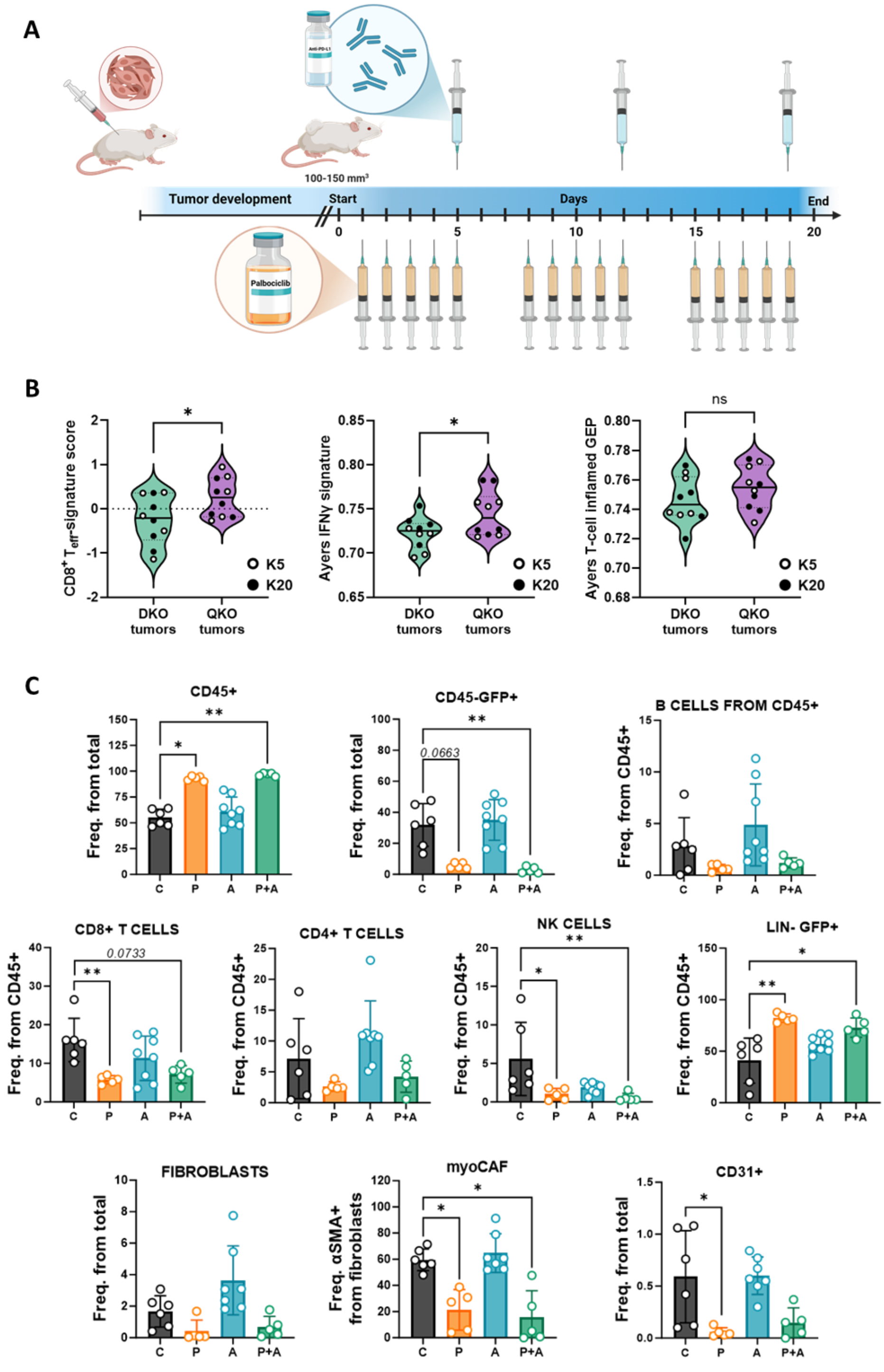

Supplementary Figure S16. Syngeneic graft BC models to evaluate palbociclib in combination with immunotherapy. A. Schematic representation of treatment regimens for monotherapy and combination therapy treatments in immunocompetent syngeneic graft BC mouse models. **B.** Analysis of three gene expression signatures to evaluate responses to neoadjuvant immunotherapy in human BC using our models. DKO and QKO tumors, classified as K5 or K20, are compared. Violin plots show individual tumors with median and interquartile ranges indicated. **C.** Analysis of alterations in tumor microenvironment populations of tumor derived from DKO-K5 cells following treatment with monotherapy and combination therapy in heterotopic tumors. Data from each individual are shown, along with the mean ± SD. Only significant differences are shown, along with trends indicated by the p-value. C = control; P = palbociclib; A = avelumab; P+A = combination palbociclib plus avelumab. LIN- = negative lineage. ns = not significant; *p-value< 0.05; **p-value< 0.01.
